# Multiplexed shRNA-miRs as a candidate for anti HIV-1 therapy: strategies, challenges, and future potential

**DOI:** 10.1186/s43141-022-00451-z

**Published:** 2022-12-28

**Authors:** Jyotsna Jai, Deborah Shirleen, Christian Hanbali, Pamela Wijaya, Theresia Brigita Anginan, William Husada, Muhammad Yogi Pratama

**Affiliations:** 1 Department of Biotechnology, Indonesia International Institute for Life-Sciences (i3L), Jakarta, Indonesia; 2 Department of Biomedicine, Indonesia International Institute for Life-Sciences (i3L), Jakarta, Indonesia; 3 Division of Vascular Surgery, Department of Surgery, New York University Medical Center, New York, USA

**Keywords:** HIV-1 therapy, Gene therapy, Gene silencing, RNA interference, shRNA, miRNA, siRNA, Multiplexed shRNA-miRs

## Abstract

The spread of HIV is on the rise and has become a global issue, especially for underdeveloped and developing countries. This is due to the fact that HIV majorly occurs asymptomatically and is implausible for early diagnosis. Recent advances in research and science have enabled the investigation of a new potential treatment involving gene-based therapy, known as RNA interference (RNAi) that will direct gene silencing and further compensate for natural variants and viral mutants. Several types of small regulatory RNA are discussed in this present study, including microRNA (miRNA), small interfering RNA (siRNA), and short hairpin RNA (shRNA).

This paper examines the mechanism of RNAi as a viable HIV therapy, using a minimum of four shRNAs to target both dispensable host components (CCR5) and viral genes (Gag, Env, Tat, Pol I, Pol II and Vif). Moreover, a multiplexed mechanism of shRNAs and miRNA is known to be effective in preventing viral escape due to mutation as the miRNA develops a general polycistronic platform for the expression of a large amount of shRNA-miRs. Several administration methods as well as the advantages of this RNAi treatment are also discussed in this study. The administration methods include (1) ex vivo delivery with the help of viral vectors, nanoparticles, and electroporation, (2) nonspecific in vivo delivery using non-viral carriers including liposomes, dendrimers and aptamers, as well as (3) targeted delivery that uses antibodies, modified nanoparticles, nucleic acid aptamers, and tissue-specific serotypes of AAV. Moreover, the advantages of this treatment are related to the effectiveness in silencing the HIV gene, which is more compatible compared to other gene therapy treatments, such as ZFN, TALEN, and CRISPR/Cas9.

## Background

Approximately 36.7 million people around the globe live with Human Immunodeficiency Virus (HIV) as well as its clinical manifestation of acquired immunodeficiency syndrome (AIDS), with 95% of the cases predominantly occurring in developing countries [1]. At present, around one million lost their lives due to issues associated with HIV worldwide, while there is still no definitive cure for HIV infection, despite the advance of modern technology in the field of infectious medicine. This explains why research surrounding HIV treatment is of utmost essential, in order to improve the survival of individuals with HIV as well as to minimize the spreading of the virus [1].

Although combinatorial antiretroviral therapy (cART) is capable of potently suppressing HIV-replication as well as delaying the onset of AIDS, the phenomenon of viral mutagenesis enables viral escape from these drugs [2]. Due to this drawback, new biological therapeutics are currently under research. One of the prime example  is gene therapy approach that uses RNA interference (RNAi) to silence viral expression or host mRNA targets essential for HIV-1 infection and replication [3]. The primary advantage of utilizing RNAi is that it compensates for viral mutants as well as natural variants, thus increasing the quantity of therapeutic targets way more than the capabilities possessed by cART [4]. Therefore, this review highlights the use of RNAi-mediated silencing as a treatment for HIV-1 infection.

## Pathology of HIV infection in human cells

The early steps of HIV-1 infection involve numerous interactions between various viral proteins and host immune cells. The surface glycoprotein of the mature HIV (gp120) first binds to a receptor found on the host cell referred to as cluster of differentiation (CD4) [5]. Attachment to CD4 molecules with the aid of gp120 results in a conformational change to the two, which ultimately leads to the opening of another site for gp120, for the purpose of easing the binding process with the co-receptor, specifically chemokine receptor 5 (CCR5) or chemokine receptor 4 (CXCR4 or fusin) [1, 6]. The binding of gp120 to CD4 as well as to the co-receptor will trigger a second conformational change begin in gp120 and then immediately in gp41 [1, 7]. In turn, this conformational change in gp41 enables the fusion of HIV-1 envelope with the cell wall, ultimately allowing the virus’ capsid to enter into CD4-positive cells.

**Fig. 1:**
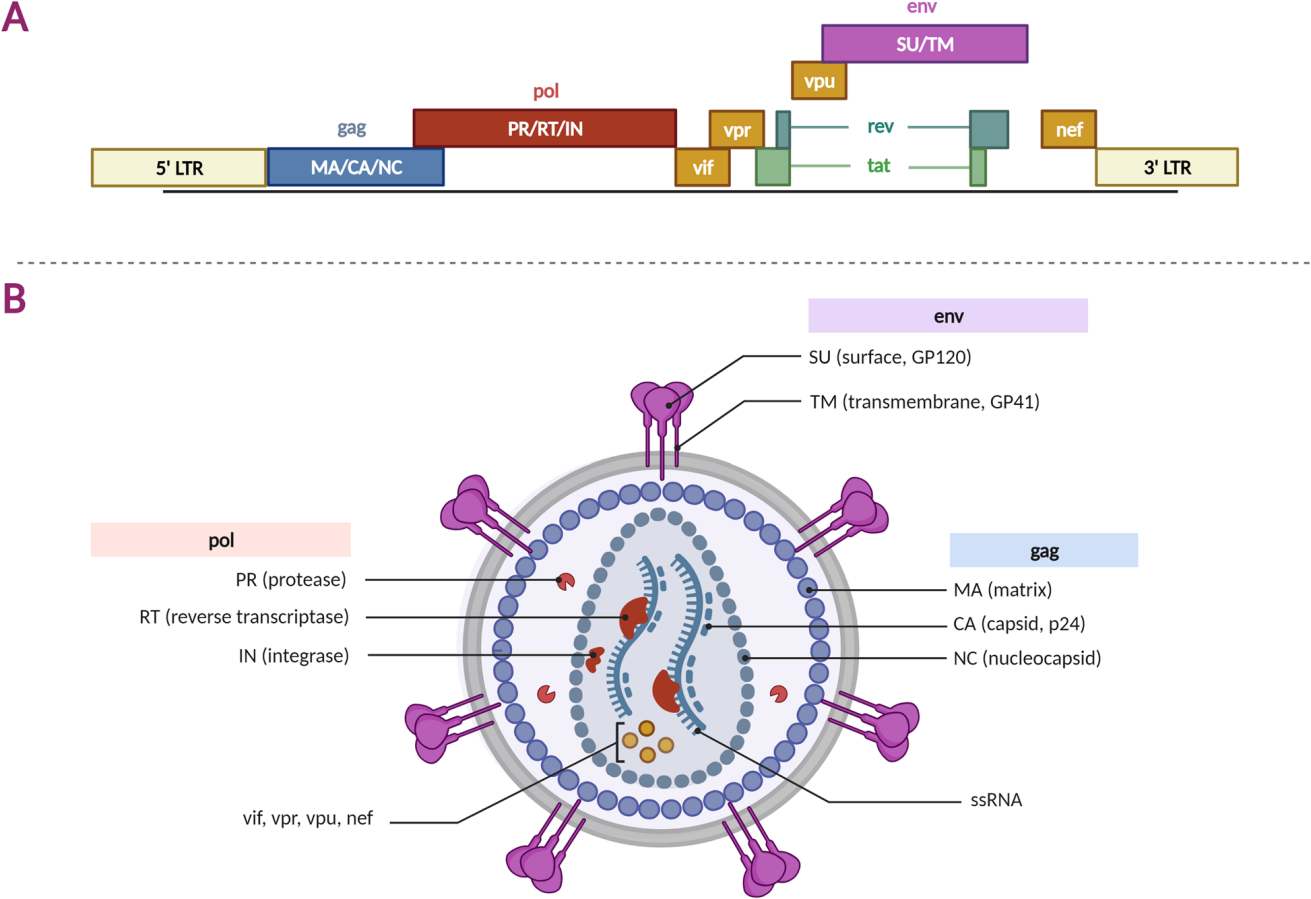
The structure of HIV-1 virus. HIV-1 virus consists of several essential genes, including long terminal repeats (LTR), group-specific antigen (gag), DNA polymerase (pol), viral infectivity factor (vif), viral protein u (vpu), envelope (env), and negative regulatory factor (nef). **a** shows the HIV-1 genome, while **b** shows the HIV-1 virion

**Fig. 2:**
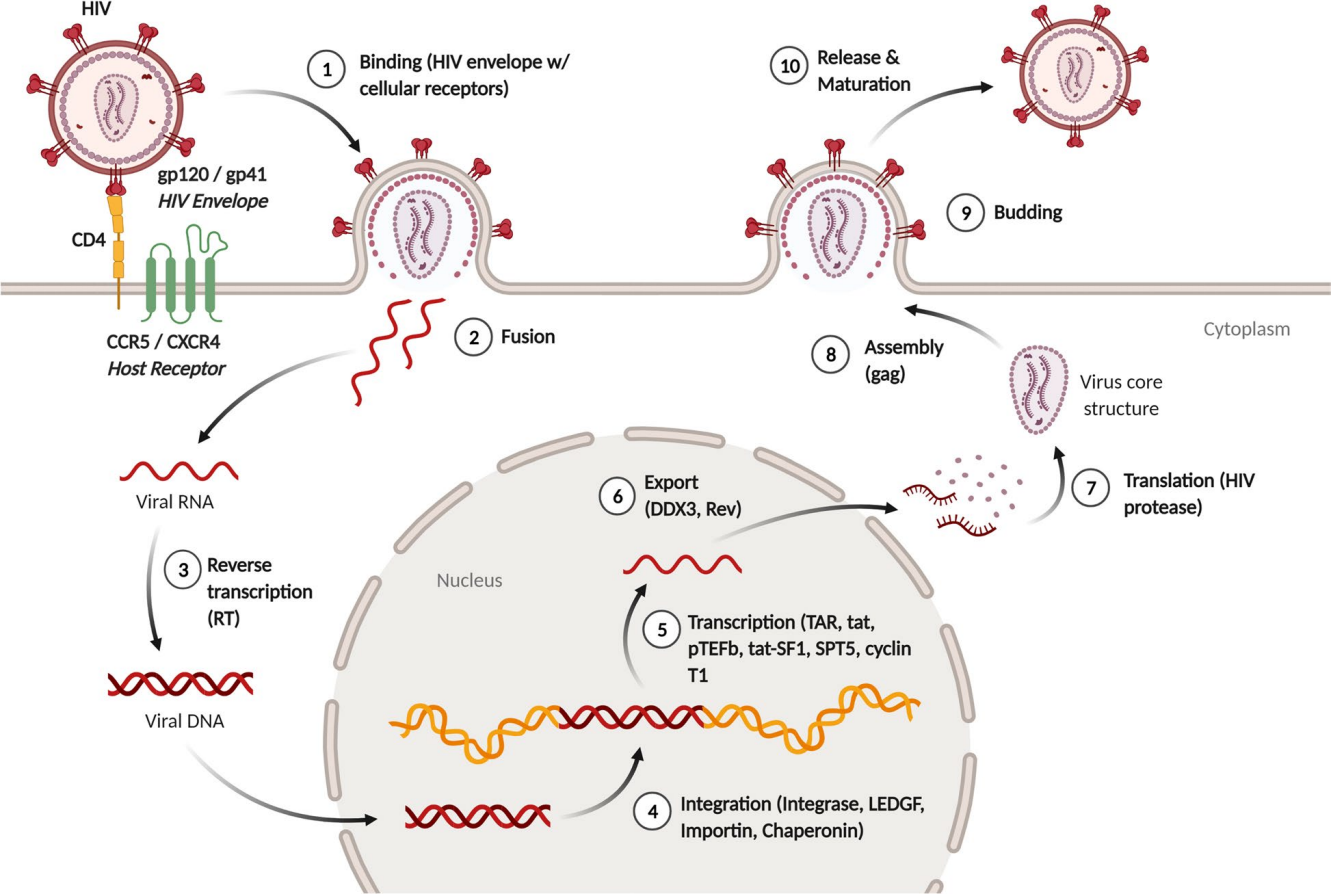
HIV-1 replication cycle. The replication cycle involves several mechanisms where the HIV virus binds with the host receptor and inserts the viral RNA inside the host cells. Reverse transcriptase is used to synthesize viral DNA which would be integrated and transcribed inside the nucleus. Afterward, the mechanism is continued by a post-translational modification within the ribosome which would result in a complete viral structure. The complete viral structure is now ready to be assembled and released from the cell to weaken the body’s immune response and lead to the onset of AIDS. Some viral factors are necessary for these processes, including trans-activation response element (TAR) and trans activator of transcription (tat). Not to mention that several host factors also help these processes, such as positive transcription elongation factor B (pTEFb), tat-specific factor 1(tat-SF1), a transcription factor encoded by SUPT5H (SPT5), lens epithelium-derived growth factor (LEFDGF), dead box RNA helicase (DDX3), and regulator expression of virion proteins

Fusion between the virus as well as cellular membranes will result in viral capsid translocation into the cytoplasm. This will cause the endosome to take up the virus’ capsid and altering  the pH value inside the phagosome, thus releasing the materials contained in the capsid into the cytoplasm [1, 8]. Furthermore, the cytoplasm serves as reverse transcriptase (RT) activation site. HIV RT will then transcribe the single-stranded HIV RNA genome into a complementary DNA (cDNA) [1]. Simultaneously, the RNA strand undergoes enzymatic degradation by RNAse H, which is subsequently followed by the action of DNA-dependent DNA polymerase activity of RT, leading to the alteration of single-stranded cDNA into double-stranded DNA (proviral DNA) [1, 9]. The proviral DNA that was formed is transported into the nucleus of the cells with the help of nucleopores in the form of a complex that contains the integrase (IN), its host factors (LEDGF, Importin and Chaperonin) as well as proviral DNA that is linear or circular in shape [10]. Then, integrase and its host factors insert the proviral genome into the human host cell genome through a randomized manner. Post-integration, transcription of the virus occurs. This step is mediated by viral factors (TAR and tat) as well as host factors and later exported to the cytoplasm (pTEFb, cyclin T1, SPT5, tat-SF1) [4]. Then, the virus undergoes a translation process and post-translational modifications that are mediated by the viral protease. Finally, these proteins undergo processing and packaging into new viral particles [1]. The structure and organization of the HIV genome is shown in Fig. 1 and the HIV-1 replication cycle is shown in Fig. 2.

## RNA interference (miRNA, shRNA, and siRNA)

RNA interference (RNAi) is a natural cellular mechanism initially identified in 1998 by Craig Mello and Andrew Fire [11]. It was described that the majority of eukaryotic cells utilize the RNAi pathway to target foreign double-stranded RNA in several organisms and serve as a predominant defense mechanism against different pathogens [12]. Furthermore, the RNA silencing pathway acts as a gene therapy tool by delivering artificial RNAi in the form of small RNA duplexes, namely microRNA (miRNA), short-interfering RNA (siRNA) as well as short-hairpin RNA (shRNA) [13]. Although siRNA and shRNA function through a similar RNAi pathway, additional processing is required when working with shRNA. As the name suggests, shRNA differs from siRNA due to the presence of a single strand of RNA possessing two complementary tails joined together by a hairpin loop [14]. Another difference is that shRNA is commonly transcribed from sources such as viral vectors or plasmid DNA located in the nucleus, while siRNA is often manmade [12]. The mechanism of the RNAi pathway is accomplished through the multi-step processing of double-stranded RNA (dsRNA). Firstly, dsRNA or primary miRNAs (pri-miRNAs) are cleaved by a ribonuclease (RNase) referred to as Dicer in order to form short dsRNA fragments which are called siRNA and miRNA, respectively. These fragments are loaded onto an RNA-induced silencing complex (RISC), which removes the non-targeting (passenger strand) and retains the targeting (guide strand) from the double-stranded fragments [15]. Then, the mRNA sequence which is complementary to that of the siRNA and the miRNA guide strand, is located by the RISC complex. The binding of either siRNA or miRNA and mRNA target in the RISC complex will lead to gene silencing via mRNA degradation and post-translational repression [4]. Although siRNA and miRNA pathways have biological similarities, the fundamental difference is that miRNAs are generally not 100% complementary to the target sequences, while siRNAs exhibits perfect complementarity [12]. This results in miRNAs inducing translational repression or targeted degradation, with siRNAs directing targeted cleavage [16]. Another difference between the two is that the biogenesis of miRNAs requires the cleavage of pri-miRNAs to precursor miRNAs (pre-mRNAs), before being exported to the cytoplasm, while the biogenesis of siRNA does not require this step [12].

**Fig. 3:**
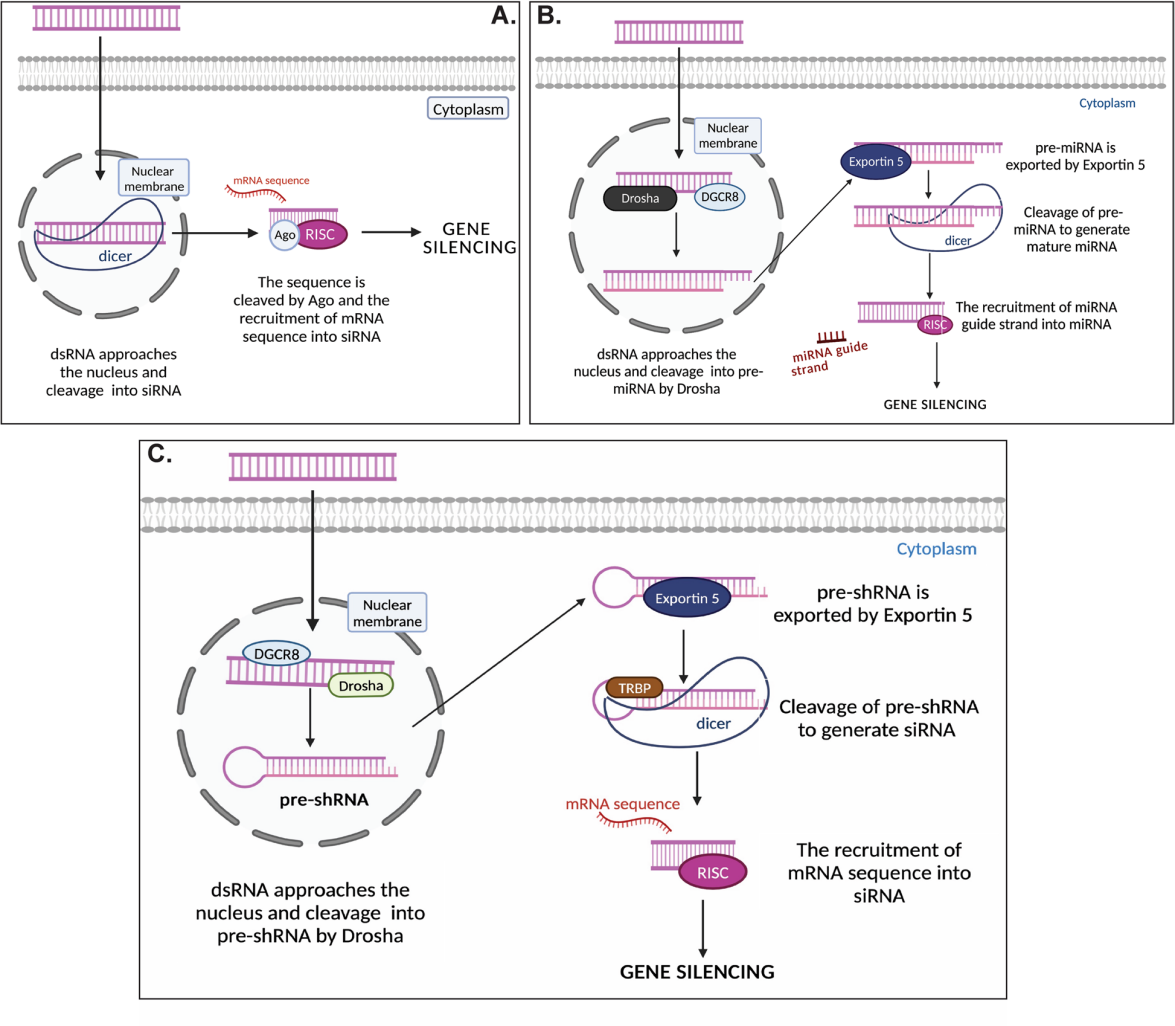
Biogenesis of RNA Interferences. **A** Biogenesis of siRNA. dsRNA of the pathogen approaches the nucleus, where it is cleaved into si-RNA. In the cytoplasm, siRNA is loaded onto RISC and undergoes cleaving by Ago protein. Then, mRNA complementary to the siRNA binds to it, leading to gene silencing. **B** Biogenesis of miRNA. Genetic material (dsRNA or pri-miRNA) of the pathogen approaches the nucleus, where it is cleaved by DROSHA in combination with DGCR8 into pre-miRNA. In the cytoplasm, the pre-miRNA is cleaved by DICER to form mature mi-RNA. Thereafter, it is loaded onto a RISC, where it binds to a complementary mRNA strand to allow gene silencing. **C** Biogenesis of shRNA. Inside the cell, dsRNA of the pathogen approaches the nucleus, where it is processed by DROSHA in combination with DGCR8 and forms pre-shRNA. In the cytoplasm, DICER in complex with TRBP cleaves pre-shRNA to siRNA. It is then loaded onto a RISC, which it binds to a complementary mRNA strand to allow gene silencing. dsRNA, double-stranded RNA; siRNA, small interfering RNA; RISC, RNA-induced silencing complex; Ago, Argonaute; mRNA, messenger RNA; pri-miRNA, primary microRNA; DGCR8, DiGeorge syndrome critical region 8; pre-miRNA, precursor miRNA; pre-shRNA, precursor Short hairpin RNA; TRBP, transactivation response element RNA-binding protein

Contrary to miRNA and siRNA, shRNAs are synthesized in the nucleus of cells that are either transfected or transduced and generally form hairpin-like structures, which comprises a stem region of sense and antisense strands that are joined together by unpaired nucleotides, that eventually result in a loop structure [14]. Additionally, shRNAs are transcribed by enzymes called RNA polymerase II or III, which ultimately depends on the promoter driving their expression [4]. Unlike siRNA, shRNA requires further processing. Before further processing, shRNAs are initially processed by Drosha (a ribonuclease III enzyme) along with its dsRNA-binding partner DGCR8 to form substances referred to pre-shRNAs [17]. These substances are then transported to the cytoplasm with the aid of a protein called exportin 5 [16]. Once in the cytoplasm, the pre-shRNA molecules can be converted into siRNAs by cleaving with dicer and transactivation response element RNA-binding protein (TRBP), an RNA-binding cofactor of dicer complexes particularly in human cells. Fareh et al. [18] suggested that TRBP is capable of increasing the RNA-binding affinity of dicer, thus enhancing the accuracy of the cleaving process. As a result, the hairpin structure is removed from the pre-shRNA molecules to create double-stranded siRNA molecules. These active siRNA molecules are then loaded onto the RISC complex for gene silencing [14]. The biogenesis of RNA interferences are summarized in Fig. 3A–C.

## Mechanism of ShRNA-mIRs interference treatment

Given the HIV-1 genome’s enormous proclivity for mutations, any successful treatment strategy would involve several shRNAs emitting both disposable host factors and viral genes, preferably simultaneously [19, 20] developed a biologically relevant stochastic model with parameters that mimic in vivo HIV infection within a 3D matrix, which aims to evaluate the efficacy of multiple shRNA gene therapies in silico. Their results show that when the shRNA number is set to four, it develops resistance in a mixed population of cells and improves overall efficacy effects that lower numbers of shRNA do not possess. Thus, to overcome the viral escape, a combination of minimum four shRNAs is required, considering that these four shRNAs correspond to each of the circulating hundreds of viral variants and the viral quasispecies found in patients [21]. If seven shRNAs are expressed at the same time, it is possible to cover all HIV-1 strains by guaranteeing that a minimum of four shRNAs are active against each virus [22]. By multiplexing more shRNA, more targets can be knocked down due to the interaction of multiple shRNAs [23].

HIV-1 major entry receptor is CD4 with CCR5 and CXCR4 as the major co-receptors [24]. However, CD4 cannot be the targeted cells for RNAi due to its dependency to T cell receptor (TCR) for the activation process [25]. As a consequence, its disruption can independently promote immunodeficiency in the host system [26]. Hence CCR 5 would make a good target, since CCR 5-tropic virus causes the primary infection of HIV-1 and has 50% less chances of mutation in the infected individuals, and if it happens to mutates, mutation in CCR 5-tropic virus was previously reported to be unthreatening [19].

Viral genes are also essential as a target for effective treatment. Several regions of the HIV-1 structure that can be set as the potential RNAi treatment target include *Gag, Pol, env, tat, rev,* and *nef* [19]. All these regions are necessary for proteolytic processing, transcription and integration, receptor binding and fusion, RNAi modulation, reverse transcription and immune modulation, respectively [4]. However, mutation frequently occurs in the virus life cycle which sometimes causes less effective treatment targeting on the viral genes [27]. Nevertheless, a study conducted by [28] suggests that gag/pol siRNA sequence is highly conserved in HIV-1. Therefore, these regions can be set as the most effective RNAi treatment targeting in which the viral assembly and infection process can be hindered due to the disruption of structure and enzymatic proteins of HIV-1.

Designing the multiplex shRNAs is the next step after determining the effective targets. The shRNA-miRs are designed in such a way that it targets CCR5 and the highly conserved regions in the viral genome, specifically: Gag, Env, Tat, Pol I, Pol II and Vif transcripts [22]. The purpose of silencing the CCR5 gene is to interfere with its translation, thus in consequence making the CD4 cells have a lesser amount of CCR5 receptor protein, which means fewer probability of the gene opening up to HIV-1, while the purpose of silencing the viral genomes is simply to inhibit its replication [29]. Using artificial miRNA clusters, a general polycistronic platform is developed to express large numbers of shRNA-miRs using minimal flanking sequences from different endogenous miRNAs backbones to express individual shRNA-miRs, but without decreasing the functionality of the miRNA [30]. The native miR stem–loop is converted with HIV-1 targeting shRNA sequences, each with 30-nt flanks for all miRNA backbones to ensure proper Drosha processing in vivo [22].

**Fig. 4:**
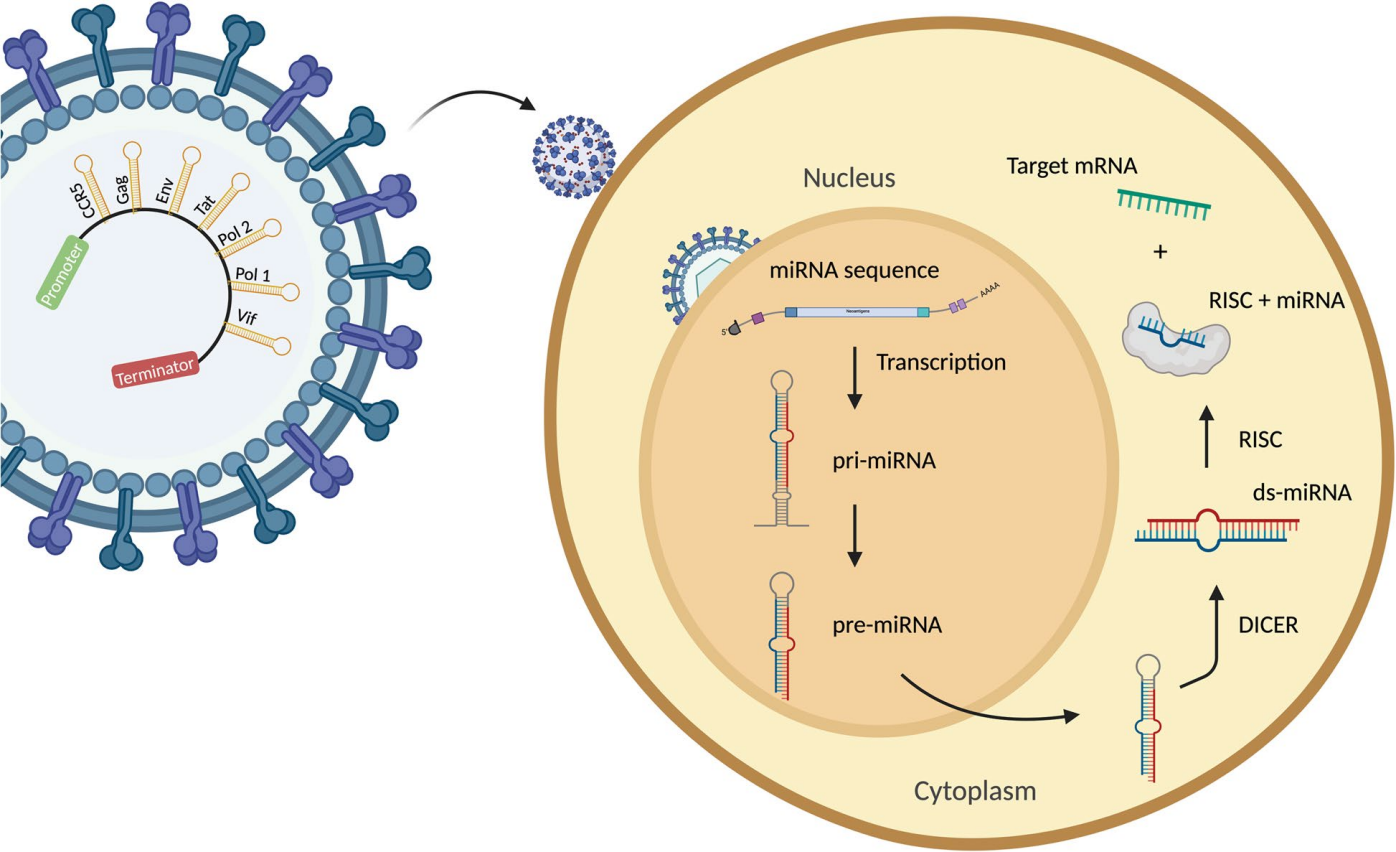
The schematic view of multiplexed shRNA-miRs (note that the amount and combination of shRNA are flexible) and the mechanism of it in silencing genes. Env-pseudotyped lentiviral vector containing multiplex shRNA-miRs integrates with CD4 T cell, transducing the miRNA-based shRNA into the nucleus. It is then transcribed into primary miRNA that is further cut into precursor miRNA and being transported out to the cytoplasm. The DICER cuts its loop head, and the produced double-stranded miRNA is unwounded by the RISC into a single strand that guides the RISC to cut the target mRNA. Target mRNA will be degraded and results in a gene silencing

The multi shRNA-miRs are delivered via HIV-1 envelope-pseudotyped lentiviral vector, allowing resting CD4 T cells to be effectively transduced and ensuring that HIV-1-susceptible cells are primarily targeted [22]. Regardless of the combination amount, The shRNA with pri-miRNA processing signals produced under the promoter(s) *Pol III* or *Pol II* or CMV promoter, are processed in the same way as natural endogenous miRNA: processed by Drosha/DGCR8 to a smaller precursor shRNA-miRNA, which is ready to be transported out of the nucleus [22, 31]. In the cytoplasm, dicer recognizes the loop of shRNA and converts it to siRNA molecule, following in which its stem is further cut, forming a short ds-miRNA [32]. Then, the Ago protein within the RISC binds with it to unwound the strand and release the other strand. The messenger RNA of a target gene which is partially complementary to the sequence of the miRNA then guides the RISC complex to bind to the specific location on mRNA by enabling base pairing [12]. Targeting via microRNA then leads to a silencing mechanism by cutting the mRNA into two halves by the proteins on the RISC complex, then the mRNA will be further degraded by the cell [14]. We summarized the mechanism of shRNA-miRs gene silencing in Fig. 4.

According to a study conducted by Applegate et al. [20], infection levels treated by four and six shRNA were reduced by 60%, while it is estimated that using seven shRNA will provide up to 87% coverage for all known strains and 100% coverage for clade B subtypes, [21]. Unfortunately, only a maximum of six shRNAs has been successfully multiplexed up to this date, while the use of seven shRNAs is still undergoing trials on humanized mice [22]. A possible hindrance for clinical trials is due to the drawbacks of delivery strategies and the possibility of viral mutation. Several studies conducted on a specific HIV-1 mutation (the CR5∆32 mutation), which is a 32-base-pair deletion in CCR5, have shown that individuals with this mutation are capable of exhibiting either partial heterozygous) or complete (homozygous) protection from infection by HIV-1 [6, 33–36]. It interferes with the ability of the virus to infiltrate cells of the immune system, because the mutation leads to the decreased and dysfunctional expression of CCR5 receptors [37]. In fact, the only documented case of an HIV-infected individual completely clearing it was the one who was initially HIV-positive and was diagnosed with myeloid leukemia. Upon receiving a bone marrow transplant from a homozygous donor for the same mutation, no detectable virus was found for more than eight years post-transplant [38–41].

A previous study conducted by Ledger et al. [42] involving the use of a short hairpin RNA to CCR5 (sh5) as a possible anti-HIV agent, showed that the therapy was able to suppress the expression of CCR5 in peripheral blood mononuclear cell (PBMC) to levels similar to that observed in individuals who are heterozygous for the CCR5∆32 mutation. This suggests that sh5 did not possess a significant effect on PBMC cells infected by HIV strains that are inefficient in utilizing CCR5, in comparison with the strong protection in CCR5 cells infected with the same HIV strain. Therefore, HIV strains incapable of using CCR5 as a target receptor, and do not infect CXCR4, can employ other receptors to attach to CD4+ cells [42]. Moreover, several other studies identified that a change in tropism was found in a patient who underwent transplantation with stem cells that were homozygous for the CCR5∆32 mutation, from a dominantly CCR5-tropic HIV-1 to a CXCR4-tropic HIV-1, [43, 44]. Since the treatment mechanism reviewed here involves the employment of CCR5 as a target, these findings can negatively affect this type of therapy. In the case of mutations of the HIV-1 strain, the therapy proposed can be deemed effective, as this combination of multiple shRNAs targets various vital regions in the HIV-1 RNA genome, which are generally considered to remain conserved in all subtypes of HIV-1 [45].

## Methods of treatment delivery

**Fig. 5:**
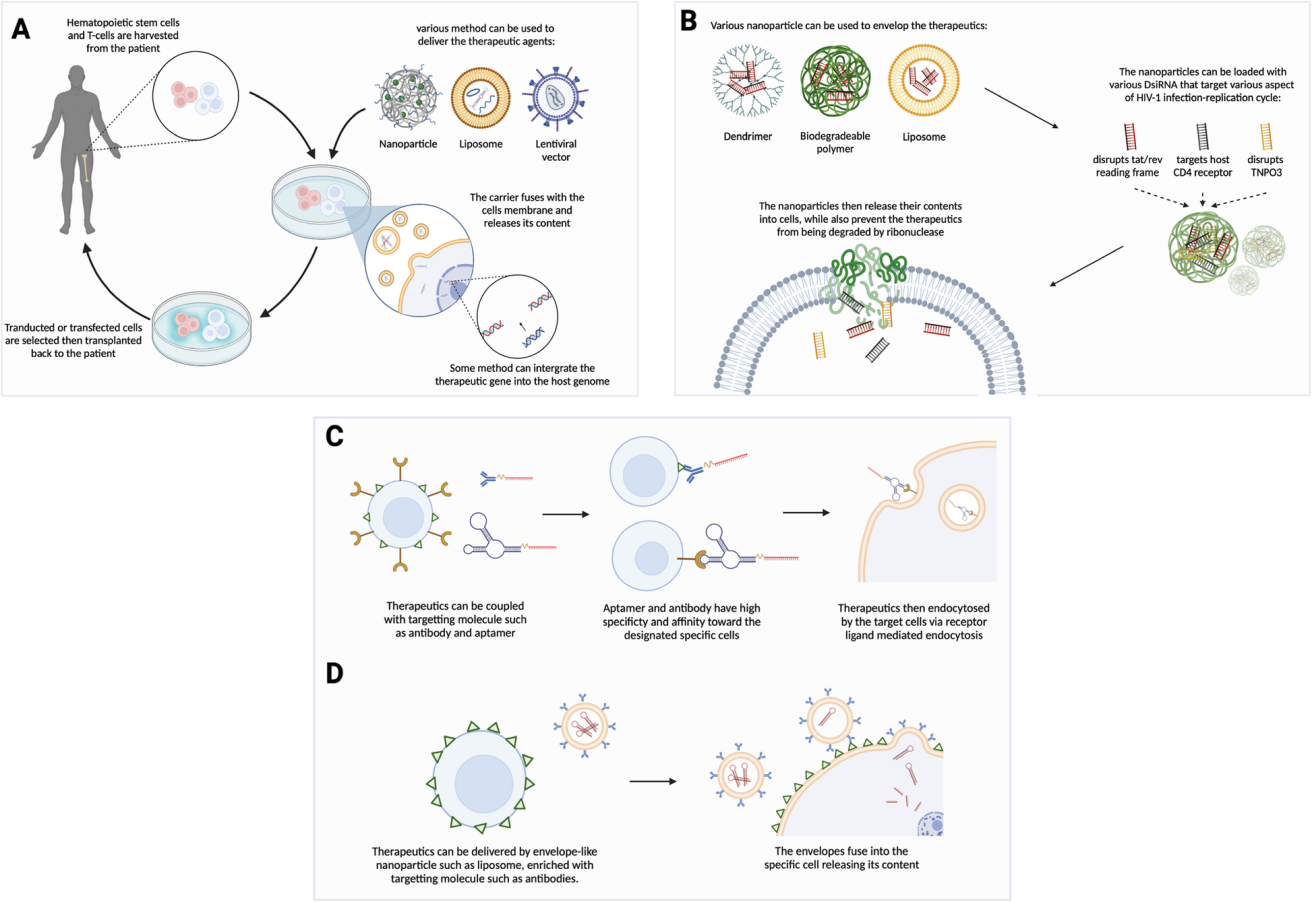
Possible methods of multiplexed shRNA-miRs delivery. **A** Ex vivo administration of therapeutics can be done on the patient's stem cells or T cell transplants. The cells are harvested from the patient’s tissue into a culture vessel, then subjected to transfection by using liposomes or nanoparticles; or transduction by using viral vectors such as adenoviral vectors or lentiviral vectors. Lentiviral vectors can integrate the therapeutic gene into the host genome. **B** Non-specific in vivo administration can utilize dendrimers, polymers, and liposomes to encapsulate the therapeutics. Various DsiRNA can also be added into the construct along with the CCR5 knockout siRNA. As the nanoparticles attach to cells, they can fuse with the cell membrane and release their content into the cell. **C** Therapeutics can be coupled with antibodies or aptamers to target specific cells. The therapeutics can latch to the cell receptor and enter the cell by receptor-ligand mediated endocytosis. **D** Envelope-type nanoparticles such as liposomes, dendrimers, and polymers can be enriched with targeting molecules to convey the siRNA to specific cells. The nanoparticles then can fuse with the cell membrane and deliver therapeutics while also preventing the siRNA degradation by ribonucleases

Several methods of delivery readily available had been tested in several studies; RNAi-based treatments can be administered as RNA in form of siRNA or shRNA, DNA in form of plasmid or mini circle, or using a viral vector. However, few factors should be considered before administering the therapy, such as cell type uptake, immunogenicity, and specificity [46]. Delivery reagents can be tailored to target specific and non-specific cells, while the targeted delivery has much more advantage over the non-specific counterpart [4]. The targeted delivery can reduce side effects while also increasing the efficiency of therapeutic conveyance toward the targeted cells. One of the RNAi-based agents that have reached clinical testing for HIV/AIDS uses an ex-vivo approach, in which the delivery is performed using a lentiviral vector targeting specific cells: CD4+ T cells or CD34+ HPCs [47]. Alternatively, in vivo delivery can use non-specific delivery system which utilizes non-viral carriers such as aptamers, dendrimer, and liposomes. The possible methods of delivery are summarized in Fig. 5.

### Ex vivo method

The ex vivo approach of RNAi delivery has several methods to incorporate therapeutic vectors including viral vectors, nanoparticles, and electroporation [48]. Based on the previous report [49], the most commonly used methods is the viral vectors, which use transduction process to encode the shRNAs into stem cells such as hematopoietic stem cells or T cells using modified viruses (Fig. 5A). The transduction process will then form new stable cell lines which are also resistant to the transfection. The viral vectors that are mostly used in this method are derived from either adenovirus, adeno-associated virus (AAV), or lentivirus [49].

The integrative characteristic of a virus must be taken into account when using the virus as the viral vector material. For example, lentiviruses are integrative; which means the RNA delivered by the virus will be integrated with the host genomic DNA [50]. Despite the insertion position being semi-random within the gene, the lentiviral vector does not share the propensity to trigger insertional oncogenesis like a gammaretroviral vector, unlike other gammaretroviruses [50]. On the other hand, adenoviral vectors (AAV), which are a derivative from ssDNA viruses are mostly non-integrative; albeit their non-integrative characteristic, AAV could integrate limitedly at a specific locus within the cellular genomic DNA [51].

Various non-viral approaches such as using liposomes and other nanoparticles to deliver therapeutic plasmid had been put into tests, but these approaches are inefficient in delivering transcripts into the T cells [4]. Transcripts delivered by these non-viral approaches are non-integrative with the host gene and have lower persistence inside the host, thus limiting their therapeutic value [51].

DiGiusto et al. reported the first clinical demonstration of RNAi usage against HIV-1 to examine the therapy safety and feasibility [52]. The test subjects were HIV patients that have developed HIV-related lymphoma, whose CD34+ HPC were transduced with a self-replicating and self-inactivating lentivirus. The lentivirus had been modified to contain a tat/rev shRNA, a TAR decoy, and a CCR5 ribozyme [53]. The patients then have lentiviral transduced CD34+ HPCs transplanted to treat the lymphoma and HIV/AIDS. The treatment was well tolerated, and no drug-related side effects were detected during the course of the treatment. The therapy also showed expected therapeutic effects-shRNA and ribozyme component expression were persistently detected in the four patient’s peripheral blood and bone marrow for no less than 8 months, with a period of more than 3 years observed in one patient [52]. The trial showed that the therapy was applicable and practical, but the efficacy of the therapy is finite and not everlasting.

### Non-specific in vivo delivery method

To deliver the HIV-1 siRNAs, various nanoparticles such as liposomes, polymers, and dendrimers, have been used to encapsulate the siRNAs [51, 54, 55]. For example, a dendrimer named G5 PAMAM (generation 5 polyamidoamine) armed with three anti-HIV-1 DsiRNAs, is able to suppress the replication of HIV-1 in a humanized mouse model [54]. Yan et al. [55] mentioned that this cocktail strategy could be combined with several other DsiRNAs: a DsiRNA target to disrupt the HIV-1 tat/rev overlapping reading frame, a DsiRNA that target the host CD4 receptor, and a DsiRNA that disrupts Transportin-3 (TNPO3) which is a HIV-1 nuclear entry facilitating host factor. Using a biodegradable polymer to encapsulate the CCR5 siRNA, the nanocapsule will protect the siRNA from being degraded by ribonuclease, but by delivering the siRNA after entering the cell. This way of delivery has shown a higher level of CCR5 gene knockout compared to the other liposome-based delivery systems [55] (Fig. 5B).

### Targeted delivery method

Targeted delivery approaches incorporate the usage of antibodies, modified nanoparticles, nucleic acid aptamers, and tissue-specific serotypes of AAV (Fig. 5C, D). All of the approaches are non-integrative and have their own set of challenges that must be considered to make it a suitable and effective delivery method. For example, for the gene therapy, while AAV is an effective vector candidate, its space for exogenous genes is limited and it also possesses immunogenic properties which only allow the AAV to be utilized as a single-time delivery agent [56].

Interestingly, the cell-specific nanoparticles approach could use targeted lipid nanoparticles, antibodies, and aptamers to be delivered as therapeutics adjunction. Although these particles are minuscule, they have to deal with multiple hurdles such as cell membrane and endosome to convey the siRNA to be loaded to the RNA-induced silencing complex [57]. According to Kim et al. [51], although targeted nanoparticles are commonly used as carriers for in vitro siRNA delivery; by conjugating a variety of functionalities to liposomes, it can also be applied for in vivo therapy. Those functionalities could be in a form of beads, chemical conjugates, or a specific protein. Then, a capsule will be made of a lipid bilayer envelopes the RNAi molecules, coated with targeting molecules at the outside to bind to the targeted receptor [51].

Other targeted delivery methods are selectively delivering siRNAs using antibodies and aptamer that binds to a surface receptor. Aptamers are single-stranded oligonucleotides (a short chain of repeating nucleotides) designed to attach with high affinity towards a target molecule or protein [58]. Aptamer candidates are selected using exponential enrichment, which utilizes a systematic evolution of ligands; to the point where aptamer’s specificity and binding affinity are similar to the antibodies [59]. Attached to an aptamer or antibody, the therapeutics siRNA can enter the cells by the receptor and ligand-mediated cellular endocytosis. Some of the molecule delivery targets include the CD4 receptor and HIV-1 gp120 protein. However, unlike other delivery methods, aptamers or antibodies do not form a protective coat around the siRNAs, making the siRNA susceptible to nuclease if left unmodified [59]. Due to their non-immunogenic properties, these particles can be used more than once for the same patient. Nevertheless, these particles are non-integrative and rapidly removed from the host body, meaning that the therapeutic siRNAs must be given as multiple-dose courses [60]. Song et al. [61] stated that cell-specific approach of siRNAs delivery is possible and has been done utilizing antibodies; for example, using protamine-gp160 antibody fusion protein conjugated to the gag siRNAs. Another example is poly-(D)-arginine and a CD7 antibody complexed with siRNAs that target the CCR5 co-receptor, viral genes tat and vif [51].

Other therapeutic methods such as locally applied microbicides, can be used to prevent virus transmission by loading it with siRNAs or aptamer-siRNAs. Those microbicides which contain CD4 aptamer-siRNA chimeras can be administered topically and absorbed into the genitalia mucosa layer, acting as prophylaxis for HIV-1 [59]. A previous study [62] showed that siRNA that is either conjugated with or without cholesterol could prevent viral transmission once applied topically on the skin. These types of treatments can confer long-lasting gene silencing that equates with antiviral activity [63]. However, to be a reliable HIV-1 prophylaxis, the siRNA based microbicides must have protection against HIV-1 itself, have wide target specificity against probable future viral variants, and have favorable cellular uptake and virus neutralization kinetics [4].

## Advantages of RNAi as HIV-1 treatment

The application of RNAi in the treatment of HIV-1 does have several benefits, primarily with its ability to survive under the environment of HIV-1. In general, RNAi treatment exhibits a specificity towards the target site but is sufficiently versatile in terms of viral specificity and can be controlled with the Watson-Crick base pairing interaction [64]. The fact that RNAi does not exhibit specificity towards a particular virus implies that it can facilitate several different therapeutic experiments [65]. This versatility helps to ensure the effective suppression and efficient silencing system, because despite this versatility, RNAi does exhibit high target specificity, making it easier to recognize an effective target site. Additionally, RNAi shows a relatively high survival ability in HIV-1-infected cells with shRNA-miR transduced, thus proving that shRNA-miRs possess a high viability against HIV-1 infection [22]. By making siRNA as an intermediary cell that could target tissues, it gives the advantage of minimizing the issues that can occur, including viral escape and problems related with treatment delivery, and at once improves the therapeutic index [26]. The mechanism is designed to target viral pathogens in many positions, thus decreasing the chances of viral escape. Seven target position is deemed to give a high protection against the viral escape [66]. Thus, mechanisms designed with seven targets will be very effective and safe due to the high suppression level. The expression of several different siRNAs can also improve the initial functionality and delay the emergence of treatment resistance [64]. Once the pre-miRNA binds with Ago2 protein, it will reduce the interference with cellular miRNA function as well [26].

RNAi mechanisms possess various benefits when compared to the other techniques. For example, ZFN, TALEN, and CRISPR/Cas9 are tools that exhibit gene knockout, which could result in permanent gene disruption that may result in toxicity and lead to the resistance of HIV-1 genes. These techniques are also incapable of targeting non-infected cells containing viral genes [22]. They cannot be used to mute HIV-1 genes and host factors simultanously, while RNAi also serves as a suitable mechanism for handling the HIV-genes and host factor simultaneously, while only targeting the specific receptor [22]. In turn, it does not give any resistance to the HIV-1 gene. The usage of the viral vector itself gives rise to one more benefit, that it eliminates the need for the treatment to be readmitted regularly to maintain the silencing of the genes: since it is independently replicating and thus providing an effective cure from a single treatment.

## Conclusion

Despite the existence of cARTs as a treatment for HIV-1, research for more effective therapeutics is on the rise due to the viral resistance of the currently available combinatorial drugs. The RNAi pathway is commonly used by eukaryotic cells as a defense mechanism for targeting the dsRNA of foreign organisms, and can therefore serve as an alternative for the treatment of HIV-1. The RNAi mechanism is also advantageous as it possesses a high survival rate under the conditions of HIV-1. Out of the three variants of the RNAi mechanism (siRNA, miRNA, and shRNA), shRNA in combination with miRNA (shRNA-miRs) exhibits the potential in this case due to the idea that the shRNA-miRs are designed to target CCR5 as well as the conserved regions of the viral genome. Considering the great proclivity of the HIV-1 genome towards mutations, a potential treatment would involve using several shRNA-miRs to target dispensable host factors and viral genes. Previous studies suggest that a minimum combination of four shRNAs is required to serve as a potential HIV-1 treatment. However, if seven shRNAs are expressed simultaneously, nearly all strains of HIV-1 will be covered. Unfortunately, at present, only a maximum of six shRNAs have been successfully developed, with seven still undergoing trials through the use of humanized mice. The ex vivo method where the bone marrow is transduced with a lentivirus vector is deemed to be the most effective administration method so far due to its success during clinical trials. Nevertheless, the effect of this administration method has been temporary, thereby concluding that further research in the in vivo experiment is still required to devise an effective method to deliver the HIV-1 treatment.

## Data Availability

Not applicable.

## Abbreviations

AAV: Adeno-associated virus
AIDS: Acquired immunodeficiency syndrome
AUS: Australia
Ago protein: Argonaute protein
Ago2: Argonaute RISC catalytic component 2
cART: Combinatorial antiretroviral therapy
CCR5: Chemokine receptor 5
CD4: Cluster of differentiation 4
CD7: Cluster of differentiation 7
CD34+: Cluster of differentiation 34 positive
cDNA: Complementary DNA
CMV: Cytomegalovirus
CRISPR/Cas9: Clustered Regularly Interspaced Short Palindromic Repeats/Cas9
CR5∆32: Chemokine receptor 5 delta base pair 32
CXCR4: Chemokine receptor 4
DsiRNAs: Dicer-substrate short interfering RNAs
DDX3: Deadbox RNA helicase
DGCR8: DiGeorge syndrome critical region
dsRNA: Double-stranded RNA
Env: Envelope
G5 PAMAM: Generation 5 polyamidoamine
Gag: Group specific antigen
Gp41: Glycoprotein protruding molecular weight 41
Gp120: Glycoprotein protruding molecular weight 120
Gp160: Glycoprotein protruding molecular weight 160
HIV: Human immunodeficiency virus
HPC: High performance computing
IN: Integrase
LEFDGF: Lens epithelium-derived growth factor
LTR: Long terminal repeats
miRNA: MicroRNA
Nef: Negative regulatory factor
shRNA-miRs: Short hairpin micro RNAs
RISC: RNA-induced silencing complex
RNA: Ribonucleic acid
RNAi: RNA interference
RNAse: Ribonuclease
RT: Reverse transcriptase
PBMC: Peripheral blood mononuclear cell
Pre-mRNAs: Precursor miRNAs
Pre-shRNAs: Precursor short hairpin RNAs
Pri-miRNAs: Primary miRNAs
Pol: DNA polymerase
pTEFb: Positive transcription elongation factor B
shRNA: Short hairpin RNA
shRNA-miRs: Short hairpin micro RNAs
sh5: Short hairpin 5
siRNA: Small interfering RNA
SPT5: Suppressor of Ty homolog 5
TAR: Trans activation response element
tat: Trans activator of transcription
tat-SF1: Trans activator of transcription stimulatory factor 1
TCR: T cell receptor
TRBP: Transactivation response element RNA-binding protein
TALEN: Transcription activator-like effector nucleases
TNPO3: Transportin-3
USA: United States of America
Vif: Viral Infectivity factor
vpu: Viral protein U
ZFN: Zinc finger nucleases
